# An Integrative Neuroscience Framework for the Treatment of Chronic Pain: From Cellular Alterations to Behavior

**DOI:** 10.3389/fnint.2018.00018

**Published:** 2018-05-23

**Authors:** Jess D. Greenwald, Keith M. Shafritz

**Affiliations:** ^1^Department of Psychology, Hofstra University, Hempstead, NY, United States; ^2^Center for Psychiatric Neuroscience, The Feinstein Institute for Medical Research, Manhasset, NY, United States

**Keywords:** chronic pain, prefrontal cortex, associative learning, cognitive behavioral therapy, mindfulness meditation, functional recovery, review of literature

## Abstract

Chronic pain can result from many pain syndromes including complex regional pain syndrome (CRPS), phantom limb pain and chronic low back pain, among others. On a molecular level, chronic pain syndromes arise from hypersensitization within the dorsal horn of the spinal cord, a process known as central sensitization. Central sensitization involves an upregulation of ionotropic and metabotropic glutamate receptors (mGluRs) similar to that of long-term potentiation (LTP). Regions of the brain in which LTP occurs, such as the amygdala and hippocampus, are implicated in fear- and memory-related brain circuity. Chronic pain dramatically influences patient quality of life. Individuals with chronic pain may develop pain-related anxiety and pain-related fear. The syndrome also alters functional connectivity in the default-mode network (DMN) and salience network. On a cellular/molecular level, central sensitization may be reversed through degradative glutamate receptor pathways. This, however, rarely happens. Instead, cortical brain regions may serve in a top-down regulatory capacity for the maintenance or alleviation of pain. Specifically, the medial prefrontal cortex (mPFC), which plays a critical role in fear-related brain circuits, the DMN, and salience network may be the driving forces in this process. On a cellular level, the mPFC may form new neural circuits through LTP that may cause extinction of pre-existing pain pathways found within fear-related brain circuits, the DMN, and salience network. In order to promote new LTP connections between the mPFC and other key brain structures, such as the amygdala and insula, we propose a holistic rehabilitation program including cognitive behavioral therapy (CBT) and revolving around: (1) cognitive reappraisals; (2) mindfulness meditation; and (3) functional rehabilitation. Unlike current medical interventions focusing upon pain-relieving medications, we do not believe that chronic pain treatment should focus on reversing the effects of central sensitization. Instead, we propose here that it is critical to focus on non-invasive efforts to promote new neural circuits originating from the mPFC.

## Introduction

Recent advances in brain imaging techniques have made it possible to devise explanatory mechanisms for the development and maintenance of chronic pain. Amongst many other medical achievements, magnetic resonance imaging (MRI) is used to visualize the anatomical integrity of the spine and its associated structures. In particular, MRI has become an imaging technique used to verify clinical presentations for disc herniation. Despite this advance in the ability to visualize nervous system structures with high clarity, questions remain as to whether MRI findings coincide with clinical pain pathology and whether MRI has value in the diagnosis and therapeutic management of chronic pain (Davis et al., 2017). For example, early work in asymptomatic individuals used MRI to examine the lumbar spine (Jensen et al., 1994). Despite having no back pain, approximately 50% of the subjects showed bulges in at least one disc while roughly 25% had disc protrusions. This study demonstrated that structural abnormalities may, in fact, be present in the absence of pain symptoms. What accounts for such discrepancies between anatomical findings vs. clinical presentations? Why do some individuals with similar MRI findings report pain while others do not?

This article reviews and examines the physiological and psychological mechanisms underlying the development of chronic pain. We first describe neuronal alterations from the peripheral nervous system to the central nervous system, addressing both the spinal cord and higher cognitive brain regions. In addition, we offer a holistic mind-body approach to treating chronic pain, and end with a hypothesis to guide future investigation.

## Central Sensitization and Additional Cellular/Molecular Mechanisms

The neural correlates of chronic pain are highly complex, involving multiple structures and molecular and cellular changes within the central and peripheral nervous system. Prior work suggests that the neural correlates underlying chronic pain may be explained through the mechanism of central sensitization, referring to hyper-sensitization of the central nervous system in response to both noxious and innocuous stimuli that can result in pain (Latremoliere and Woolf, 2009; Woolf, 2011). When central sensitization takes place, prolonged peripheral nociceptive input results in the excitatory release of chemicals, triggering a transduction cascade. Multiple protein kinases phosphorylate the three ionotropic glutamate receptors, N-methyl-D-aspartate receptors (NMDAR), α-amino-3-hydroxy-5-methyl-4-isoxazolepropionic acid receptors (AMPAR) and kainite receptors, thereby increasing receptor activity and density. This process results in excitatory postsynaptic potentials reaching the dorsal horn of the spinal cord (Ballantyne, 2006; Ultenius et al., 2006; Latremoliere and Woolf, 2009). Phosphorylation of AMPAR facilitates AMPAR insertion in the synapse (Esteban et al., 2003; Galan et al., 2004). In addition to the contribution of the three ionotropic glutamate receptors, metabotropic glutamate receptors (mGluRs) contribute to both presynaptic and postsynaptic neuronal excitability (Anwyl, 2009; Niswender and Conn, 2010), often through the activation of second messenger pathways. Recent evidence also indicates that increased neuronal excitability in the dorsal horn is attributed to GABA disinhibition through the potassium-chloride cotransporter type 2, KCC2 (Kahle et al., 2014). In this process, decreased KCC2 function leads to an increase in intracellular Cl^−^ concentration, shifting the reversal potential of Cl^−^ channels in GABA_A_ receptors beyond the threshold for action potential generation. Postsynaptic neurons thus become depolarized (rather than hyperpolarized) and the resulting decrease in the overall inhibitory function of GABA_A_ receptors facilitates central sensitization (Price et al., 2009; Kahle et al., 2014).

Another mechanism for neuronal excitability is through the action of G protein-coupled receptors (GPCRs), a diverse family of cell receptor proteins that are dispersed throughout the peripheral and central nervous system. GPCRs are located on the plasma membranes and nerve terminals of sensory neurons along nociceptive pain pathways (Pan et al., 2008). Nociceptor hyperexcitability, which is believed to contribute to the transition from acute to chronic pain, is associated with a change in GPCR signaling (Dina et al., 2009). Moreover, long-term synaptic remodeling results from the dynamic processes of protein synthesis and degradation (Alvarez-Castelao and Schuman, 2015). Theoretically, maladaptive nociceptive input mediated by GPCR signaling could normalize over time as a result of protein degradation and synthesis, thereby providing hope that chronic pain symptoms may abate. Currently, however, it is difficult to quantify GPCR turnover rate (Ross, 2014), and thus, the possibility that rapid turnover of sensory receptors could potentially reverse chronic pain symptoms requires further investigation.

Persistent pain sensations are not only modulated by neurons but also by glial cells, specifically astrocytes and microglia. Increased astroglial activity increases the release of excitatory neurochemicals, such as proinflammatory cytokines and precursors to glutamate (Broer et al., 2004; Guo et al., 2007; Milligan and Watkins, 2009; Chiang et al., 2011), and can affect chronic pain by its influence on nociceptive input and the recycling of glutamate (Guo et al., 2007; Milligan and Watkins, 2009). By converting extracellular glutamate into glutamine, astroglial cells provide presynaptic neurons with the raw ingredients to continue producing glutamate, an excitatory neurotransmitter (Broer et al., 2004; Chiang et al., 2011). In addition, astroglial activity produces pro-inflammatory cytokines which, in turn, increase nociceptor activity (Zhang and An, 2007; Uçeyler et al., 2009). Microglial cell activation also mediates neuronal excitability by the reversal of the inhibitory effect of GABA. Following neuronal injury, activated microglia release brain-derived neurotrophic factor which downregulates KCC2 in the dorsal horn, thereby facilitating the process of central sensitization through GABA excitation, as described above (Coull et al., 2005; Latremoliere and Woolf, 2009; Price et al., 2009; Taves et al., 2013). Further research into the excitatory role of glial cells may provide very useful information for the treatment of chronic pain syndromes. Unfortunately, glial cells are distributed throughout the central nervous system and serve many functions. As a result, treatment targeting glial cells has a high probability of causing adverse side effects.

**Figure 1:**
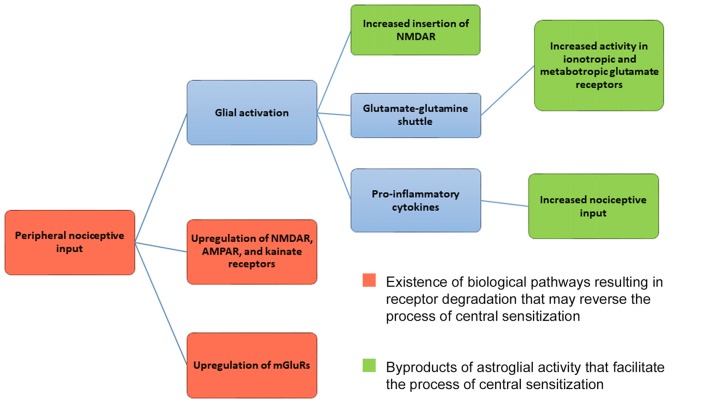
Mechanism for central sensitization. Central sensitization (central nervous system hypersensitivity) is initiated from the upregulation of ionotropic glutamate receptors (NMDAR, AMPAR, kainite receptors) and metabotropic glutamate receptors (mGluRs) in the presence of peripheral nociceptive input. As a result, neurons in the dorsal horn of the spinal cord and central nervous system respond to nociceptive input at lower thresholds, with new enlarged receptor fields, and undergo increased rates of spontaneous firing. Glial activation can further maintain the mechanisms underlying central sensitization by increasing NMDAR and AMPAR insertion in postsynaptic membranes. Glial cells release pro-inflammatory cytokines, serving as further nociceptive input. Astroglial cells also help to maintain glutamate levels via the glutamate-glutamine shuttle, which can influence both ionotropic and mGluR activity. As shown in red, ionotropic, metabotropic receptors and nociceptors are capable of being degraded. Given that degradative pathways exist, the process of central sensitization can be reversed. Activity by astroglial cells, however, may mitigate the effects of receptor degradation by upregulating and facilitating the process of central sensitization. Given that central sensitization does not reverse itself with time, it seems that astroglial activity overpowers the existence of degradative receptor pathways.

Regardless of glial cell activation and recycling of glutamate via the glutamate-glutamine shuttle, ionotropic and mGluRs undergo degradative pathways. Research has shown that degradative pathways exist for NMDAR (Scott et al., 2004; Piguel et al., 2014), AMPAR (Ehlers, 2000; Barry and Ziff, 2002), kainate receptors (Martin and Henley, 2004; Lerma and Marques, 2013) and mGluRs (Latremoliere and Woolf, 2009; Klein et al., 2015). Hence, critical receptors involved in central sensitization undergo degradation (see Figure 1). Theoretically, then, biological mechanisms already exist that can reverse central sensitization via glutamate receptor degradation. If receptor degradation were the primary mechanism by which central sensitization were reversed, chronic pain should abate with time. Yet this is not always the case. One can argue, though, that increased astroglial activity can upregulate ionotropic or mGluRs and nociceptors (see Figure 1), canceling out any putative effect of glutamate receptor degradation. This is a valid point that will require further research in the future. Given that central sensitization does not simply reverse with time, it appears that astroglial activity facilitates central sensitization at a greater rate than receptor degradation reverses the process of central sensitization (Figure 1).

Evidence suggests that central sensitization is characterized by heterosynaptic facilitation (Ji et al., 2003; Malenka and Bear, 2004; Rygh et al., 2005; Latremoliere and Woolf, 2009; Galvan et al., 2011). Central sensitization is distinct from the phenomenon known as windup, which involves homosynaptic facilitation (Latremoliere and Woolf, 2009; Woolf, 2011). Windup refers to the increased magnitude of incoming C-fiber signals at the dorsal neurons due to the increased frequency of C-fiber activation (Li et al., 1999; Campbell and Meyer, 2006), and is associated with primary hyperalgesia (increased pain at the site of injury; Ikeda et al., 2006; Sandkühler, 2007; Latremoliere and Woolf, 2009). When central sensitization has taken place, neurons at the dorsal horn respond at a lower threshold to peripheral inputs, have increased receptive fields, and have increased rates of spontaneous firing. These structural and functional alterations help to explain why patients with chronic pain experience allodynia (pain response in the absence of a painful stimulus) and secondary hyperalgesia (pain outside the site of injury; Campbell and Meyer, 2006; Latremoliere and Woolf, 2009; Woolf, 2011).

This process appears very similar to the molecular and cellular changes that occur during long-term potentiation (LTP) in cortical and subcortical brain regions (Ji et al., 2003; Latremoliere and Woolf, 2009). Considering the strong parallels between central sensitization and LTP, it is reasonable to infer that similar (although perhaps not identical) processes are occurring in spinal structures and in cortical/subcortical regions that drive the move from acute to chronic pain. Interestingly, LTP in both the amygdala (Dityatev and Bolshakov, 2005; Sigurdsson et al., 2007) and hippocampus (Whitlock et al., 2006) has long been associated with fear conditioning. Moreover, chronic pain and fear conditioning are intricately connected (Turk and Wilson, 2010; Elsenbruch and Wolf, 2015), suggesting that spinal pain-related circuits and cortical/subcortical fear circuits may undergo some of the same neural alterations (or at the very least, similar parallel processes) during the transition from acute to chronic pain. Thus, we will now describe some of the cellular and behavioral mechanisms in fear conditioning, which will assist with understanding how to better treat chronic pain.

## Pain-Related Fear and Avoidance Behaviors

Pain-related fear promotes maladaptive cognitive and behavioral responses (Vlaeyen and Linton, 2000; Turk and Wilson, 2010; Parr et al., 2012; Elsenbruch and Wolf, 2015; Vlaeyen, 2015), best characterized by a fear-avoidance model (Leeuw et al., 2007). In this model, pain perception begins with catastrophization, the belief that pain symptoms are indicative of a far worse injury, in turn resulting in pain-related fear and pain-related anxiety. Importantly, pain-related fear and anxiety are not synonymous and appear to be two distinct phenomena, as fear-induced defensive responses and anxiety-related responses are mediated by different neural circuits (LeDoux, 2015). We argue that pain-related fear can stem directly from threat perception; if an individual views an ongoing activity or present object as threatening, that individual will actively attempt to escape from that situation. Pain-related anxiety, on the other hand, can result from the anticipation of pain; if anticipating future painful events, an individual will actively avoid that event or behavior. Through both escape and avoidance, then, chronic pain patients are likely to not use the painful body area and perhaps avoid activity altogether.

How do chronic pain patients acquire pain-related fear and then in turn pain-related anxiety? These processes appear to be the direct result of fear conditioning and avoidance learning. Pavlovian fear conditioning occurs due to the association between a previously neutral stimulus and an aversive stimulus that leads to an unpleasant response (Milad and Quirk, 2012; Elsenbruch and Wolf, 2015). In the case of conditioned fear of pain, the conditioned stimulus (CS) may include both interoceptive conditioning (IC) and functional movement (De Peuter et al., 2011). Interoception, as used here, does not only refer to sensation within the viscera, but also to somatosensory and nociceptive sensation. Because pain acts as an aversive stimulus, pain-related fear can be acquired through Pavlovian fear conditioning. When this is the case, an individual with chronic pain can reverse the process through extinction of the conditioned fear (Quirk et al., 2010; Milad and Quirk, 2012). In order for fear extinction to take place, two events must take place simultaneously or in very close temporal proximity: (1) the fear association must be active while; (2) a new non-painful stimulus is introduced (Quirk et al., 2010). Importantly, extinction is not synonymous with forgetting. Rather, extinction occurs when a new association either inhibits the original fear association or alters the affective and mnemonic properties of that association (Bouton, 2004; Craske et al., 2008; Radulovic and Tronson, 2010; De Peuter et al., 2011; Kattoor et al., 2013).

Fear-associated behaviors, however, do not only arise through Pavlovian conditioning, but also through operant conditioning (Bouton and Todd, 2014; Trask and Bouton, 2014). According to the two-factor theory and related models of maladaptive fear-related behaviors, Pavlovian conditioning facilitates fear acquisition while operant conditioning results in negative reinforcement that maintains avoidance and escape behaviors over time (Krypotos et al., 2015). In standard fear-avoidance models (Leeuw et al., 2007), initial escape behaviors are negatively reinforced due to the immediate reduction of pain. Avoidance behaviors then become associated with lack of pain altogether, and this continuation of negative reinforcement prolongs the appearance of those behaviors. Of particular relevance for our theory, avoidance learning may have a greater and more enduring effect of overall threat reduction when compared with extinction (Boeke et al., 2017), and negatively reinforced escape behaviors can persist even when the initial classically conditioned CS-US pairing is extinguished (Krypotos et al., 2015). It is also possible that the persistence of an avoidance behavior can prevent the acquisition of knowledge that a CS no longer signals the presence of a noxious US, thus preventing extinction of the CS-US bond. By actively controlling when and how to use a painful body area, chronic pain patients may be inadvertently driving inherent mechanisms of threat reduction and continuing with a pattern of learned avoidance behavior that may no longer be necessary.

Three primary brain structures have been associated with the fear conditioning process: the medial prefrontal cortex (mPFC), the amygdala and the hippocampus (Phelps et al., 2004; Phelps and LeDoux, 2005; Milad et al., 2007; Herry et al., 2010; Pape and Pare, 2010). How do we know that extinction does not erase fear memories within these structures and that alternate associations are learned during extinction? Behavioral studies have shown that fear associations return after fear extinction has taken place (Morris and Bouton, 2007; Milad and Quirk, 2012), suggesting that fear memories are not erased but rather are inhibited or altered by new memories and associations. Further, it is unlikely that the cellular and synaptic changes associated with fear extinction occur as a result of long-term depression (LTD), as prefrontal LTD results in increased fear recovery after extinction (Herry and Garcia, 2002; Courtin et al., 2013). Therefore, it appears as if the primary driving mechanism for the extinction of conditioned fear is the establishment of new synaptic connections through LTP, leading to formation of new associations that dampen the effects of the original fear memory.

As mentioned above, central sensitization is a critical factor in the development of chronic pain syndromes, including (but not limited to) phantom limb pain, complex regional pain syndrome (CRPS), musculoskeletal pain and post-surgical pain (Woolf, 2011). In order for central sensitization to be reversed, there must be a downregulation of ionotropic/mGluRs and nociceptors. However, if pain relief follows a similar mechanism to the extinction of conditioned fear, then central sensitization may not need to be reversed in order for an individual to experience pain resolution. Similar to the extinction of conditioned fear, it may be possible to bypass a necessary reversal of central sensitization by establishing new neural circuits that inhibit well-established pain associations. Are these new inhibitory associations formed exclusively between the mPFC, amygdala and hippocampus as is seen in fear conditioning? There is abundant evidence that the anterior cingulate cortex (ACC) and the insula are strongly involved in the perception and integration of various pain signals (Tracey and Mantyh, 2007; Lee and Tracey, 2010; Wiech et al., 2010; Segerdahl et al., 2015). Perhaps these regions are also critically involved in the abatement of chronic pain. Future studies should address these critical questions. Moreover, it is conceivable that the default mode network (DMN), described below, may play a key mediating role in pain processing in chronic pain syndromes.

## The Default Mode Network, Attentional Network and Pain Processing

Research designs utilizing functional MRI (fMRI) to examine the functional neural correlates of chronic pain can be categorized into three primary groups: (1) pain-induction trials; (2) resting state, in which no active cognitive or sensorimotor task is performed; and (3) active tasks that require perceptual, sensorimotor, or other cognitive and behavioral processes. The latter category may also include simple motor tasks, such as finger tapping, to serve as control or comparison conditions. During a resting state, researchers hope to identify underlying brain mechanisms that persist in chronic pain patients even after a painful stimulus is removed. The brain regions showing increased activation during wakeful rest, when compared with task-active states, have been described as the brain’s DMN (Raichle et al., 2001).

Researchers frequently investigate the DMN using three processes that can be compared to one another: (1) task-free or task-independent trials in which the subject is not assigned any stimulus or task; (2) simple sensorimotor and cognitive tasks; and (3) complex sensorimotor and cognitive tasks. By comparing either task-free designs or simple sensorimotor tasks to complex cognitive tasks, researchers can examine how brain regions in the DMN change from a resting state to a task-activated state.

Structures associated with the DMN include the mPFC, medial temporal lobes (including the hippocampus) and posterior cingulate cortex (PCC; Buckner et al., 2008; Harrison et al., 2008; Greicius et al., 2009; Sheline et al., 2009). Increased DMN activity is associated with self-referential thoughts, future planning, and autobiographical memory (Gusnard et al., 2001; Buckner et al., 2008; Peeters et al., 2015). Interestingly, chronic pain is associated with dysregulation within the DMN (Baliki et al., 2008, 2014; Otti et al., 2013), and this dysregulation may help to explain the disease processes of chronic pain syndromes.

The medial frontal lobe, including the ACC, serves many different roles including error detection (Holroyd et al., 2002), conflict monitoring (Botvinick et al., 2004), and other aspects of executive functioning and cognitive control (Ridderinkhof et al., 2004; Posner et al., 2007; Alexander and Brown, 2010). It is also implicated in the storage of fear memories and retrieval of those memories (Quinn et al., 2008). Functional subregions within mPFC include its dorsal (dmPFC) and ventral (vmPFC) components. The dmPFC appears to mediate action-related activity, in part by exerting top-down control on the motor cortex and inhibiting motor output (Narayanan and Laubach, 2006). The vmPFC is heavily implicated in emotional regulation (Euston et al., 2012). Together, these regions play a critical role in both the DMN and salience network, and individual differences in autonomic reactivity may correspond to functional connectivity of mPFC to other brain areas (Jennings et al., 2016). The ACC, although not part of the DMN, is activated along with other areas of mPFC during fear appraisal, but not necessarily during fear learning (Maier et al., 2012), and is an integral structure of the salience network, along with the insula (Sridharan et al., 2008; Bonnelle et al., 2012). In the case of acute nociceptive input, the anterior insula has been shown to integrate sensory information to help determine whether or not a stimulus is painful (Wiech et al., 2010), while the contralateral dorsal posterior insula has been associated with tracking the intensity of an applied noxious stimulus (Segerdahl et al., 2015). The conversion of acute pain-induced fear associations into long-lasting memories is associated with changes in functional connectivity of mPFC that are also related to self-reported anxiety levels (Tseng et al., 2017). It is important to note that the dorsolateral prefrontal cortex (dlPFC)—long known for its role in working memory, attention and inhibition—is also critical for emotional information processing (Etkin et al., 2006; Shafritz et al., 2006; Urry et al., 2006) and pain perception (Lorenz et al., 2003; Brighina et al., 2011).

If vmPFC communicates with the ACC, a key structure in the salience network during fear appraisal, then vmPFC may be able to downregulate salient pain signals. Studies have shown that pain-related fear correlates positively with pain intensity and disability (Crombez et al., 1999; Al-Obaidi et al., 2005; Gheldof et al., 2006). In addition, individuals who claim to have a higher pain-sensitivity level display increased activation in regions of PFC and ACC in response to acute pain compared with individuals with low pain sensitivity (Coghill et al., 2003). Furthermore, acceptance-based therapies have the potential to alter activation of PFC in response to pain (Jensen et al., 2012) and to alter functional connectivity between PFC and emotion-processing regions (Young et al., 2017). Importantly, therapies emphasizing acceptance of pain are associated with clinically-meaningful symptom reduction (Vowles et al., 2007a,b; Vowles and McCracken, 2008). It is not unreasonable to suggest that pain intensity and disability in chronic pain patients can be greatly reduced through top-down control of the mPFC on the DMN, salience network, and fear association network. Thus, chronic pain patients may have the ability to alter both brain activity and connectivity by altering thoughts related to pain perception, as described in a later section of this article.

There has been much speculation as to the significance and implications of DMN activity. Mason et al. (2007) believe that activity within the DMN during task-free (stimulus-independent) thoughts is a reflection of mind wandering. Others have countered that it is impossible to decipher whether resting-state brain activity is due to stimulus-independent thoughts (mind-wandering) or due to stimulus-dependent thoughts (Gilbert et al., 2007), particularly when DMN activation is observed when a simple task is compared with a more cognitively demanding task (McKiernan et al., 2003). In order to determine the difference between mind-wandering and either IC or hyperfixation/hypervilance (two examples of stimulus-dependent thoughts), researchers can use fMRI to assess chronic pain patients while at rest and while the patients actively monitor their symptoms, perhaps by alternating blocks of rest with active pain monitoring. Such a study will help to differentiate between stimulus-independent thoughts and pain stimulus-dependent thoughts. At the moment, we can hypothesize that dysregulation of the DMN in chronic pain results from stimulus-dependent thoughts focused around IC and pain perception.

## Concerns Regarding Neuroimaging Studies of Chronic Pain

Thus far, we have highlighted the critical importance of the mPFC in the development and maintenance of chronic pain. Research on chronic pain patients, however, has revealed altered activity and cortical reorganization within other brain regions. Before we examine specific chronic pain syndromes, we must first take into consideration two confounding factors when interpreting neuroimaging studies of chronic pain. First, for pain studies that require the application of noxious stimuli, one must discern whether the fMRI results are due to somatosensory sensation or due to pain sensation. Moulton and associates (Moulton et al., 2012) found that BOLD responses might not necessarily reflect pain but rather somatosensory input and integration, because activation of somatosensory and motor cortices correlated more with heat intensity than with heat-induced pain. On the other hand, fMRI can be used to discern thermal induced pain from heat sensation and social pain (Wager et al., 2013). It is important to note that in this study, the specificity and sensitivity between thermal-induced pain and social pain was markedly lower than the specificity and sensitivity between thermal induced pain and heat sensation. Thus, when examining fMRI results, it may be harder to differentiate physical pain from emotional pain. This issue highlights the intricate relationship between physiological pain and pain perception, including social aspects of pain.

Second, individuals suffering from chronic pain have usually undergone numerous interventional treatments and have unique personal experiences owing to the specific features of their chronic pain syndrome. Both of these factors have the potential to serve as major methodological confounds and/or contribute to individual differences. Further, cognitions and emotions clearly play a significant role in chronic pain, and daily mood may affect BOLD activation when using fMRI. As a result, no two chronic pain experiences are identical and large variances from individual differences are the norm, thereby providing further difficulty in dissecting the neural mechanisms of chronic pain.

## Descending Nociceptive Pathways and Their Modulatory Role in Pain

The brainstem contains a collection of structures responsible for a descending pain modulation system, as structures within this region can produce analgesic effects and monitor nociceptive communication. Specifically, the periaqueductal gray-rostral ventromedial medulla (PAG-RVM) system allows for the bidirectional control of pronociception (nociceptive facilitation) and antinociception (nociceptive inhibition; Tracey and Mantyh, 2007; Heinricher et al., 2009). Chronic pain may arise from a dysregulation of nociceptive pathways, in particular an enhancement of pronociception or a reduction of antinociception (Heinricher et al., 2009). Within the RVM there are three types of neurons: ON, OFF and NEUTRAL cells. Evidence suggests that ON cells promote pronociception while OFF cells facilitate antinociception (Kincaid et al., 2006; Heinricher et al., 2009; Ossipov et al., 2010; Staud, 2013). Additionally, the dorsal reticular nucleus (DRt) and ventrolateral medulla (VLM) play a critical role in descending nociceptive control with the Drt facilitating nociception and the VLM inhibiting nociception (Heinricher et al., 2009). Although the PAG-RVM system combined with the Drt and VLM contribute to the development of chronic pain, these structures are both directly and indirectly modulated by the top-down influence of cortical and subcortical structures within the salience and fear networks, such as mPFC, ACC, amygdala, insula and hypothalamus (Tracey and Mantyh, 2007; Heinricher et al., 2009; Lee and Tracey, 2010). Thus, it appears that pain perception in general is modulated by higher cortical brain regions, which in turn can modify descending nociceptive pathways.

As mentioned previously, chronic pain can result from a variety of pain-related syndromes. Below, we provide neuroimaging and treatment results for a few of these syndromes, focusing upon the similarities and distinctions among the syndromes that have the potential to inform models of the brain mechanisms of chronic pain. Theoretically, these mechanisms may exert a top-down influence on the nociceptive modulation system in cases of dysregulation within the brainstem. Additional research must be conducted investigating the role of cognitions and emotions as they relate to cortical/subcortical regulation of brainstem structures, and fMRI is well-suited for that level of investigation. Such research may provide, for example, brain imaging evidence of the therapeutic effects of cognitive and acceptance based therapies in chronic pain management.

## Phantom Limb Pain and CRPS

Phantom limb pain is described as pain occurring in a lost or amputated limb due to referred sensation from neighboring bodily regions (Nikolajsen and Jensen, 2001). Symptoms of phantom limb pain include but are not limited to burning, itching, tingling, electrical sensations, cramping, and muscle spasms (Flor, 2002). The neural mechanisms resulting in phantom limb pain mirror that of central sensitization (Woolf et al., 1992; Subedi and Grossberg, 2011; Woolf, 2011). CRPS, previously known as reflex sympathetic dystrophy (RSD), is a pain disorder characterized by allodynia, swelling, dysautonomia and temperature changes that cannot be attributed to another physiological disorder (Jänig and Baron, 2002; Marinus et al., 2011). Traumatic injury has been described as one of the leading causal factors resulting in CRPS, occurring in 30%–77% of presenting patients (McBride and Atkins, 2005). These symptoms, however, may spread from one part of the body, such as the right upper arm, to another distal extremity like the lower left leg. Similar to phantom limb pain, CRPS appears to be facilitated by the process of central sensitization (Bruehl, 2010; Woolf, 2011).

Theoretically, the symptoms of phantom limb pain and CRPS should reverse through NMDAR antagonists. If central sensitization facilitates both phantom limb pain and CRPS, downregulation of NMDAR should drastically reduce symptoms. NMDAR antagonists, however, have variable effects on patients with phantom limb pain (Nikolajsen et al., 1996; Huse et al., 2001; Maier et al., 2003; Robinson et al., 2004; Schley et al., 2007) and CRPS (Koffler et al., 2007; Schwartzman et al., 2009; Pickering and McCabe, 2014). In addition, sympathetic blocks that provide a local anesthetic to block incoming pain signals are not a universally successful treatment for CRPS (Cepeda et al., 2002, 2005; Meier et al., 2009; Stanton et al., 2013).

What accounts for the maintenance of both chronic pain disorders, considering that pharmacologic intervention is of limited value? Although the primary culprit may be faulty communication between mPFC and limbic structures, other brain regions have been implicated in both pain syndromes. Phantom limb pain is associated with cortical reorganization within the de-afferated primary motor cortex and S1 (Birbaumer et al., 1997; Lotze et al., 2001; MacIver et al., 2008). Similar to phantom limb pain, CRPS is associated with cortical reorganization of the primary motor cortex (Maihofner et al., 2007; Kirveskari et al., 2010; Pleger et al., 2014) and S1 (Maihofner et al., 2003; Pleger et al., 2014), although some research suggests this is not always the case (Di Pietro et al., 2013a,b). Regardless of cortical changes in S1, there is reason to believe that such alterations do not produce pain (Gustin et al., 2012). Knecht et al. (1998) reported that cortical reorganization normally resulting from amputation of a limb does not necessarily correspond to changes in phantom limb pain perception over time. In the study, over a 4-week period, the overall extent of cortical reorganization and number of sites associated with mislocalization of phantom limb pain remained constant. Mislocalization refers to changes in the location of painful areas of the body after the application of non-noxious stimuli (touch, vibration and heat) and painful stimuli. To the surprise of the researchers, the topography of mislocalized pain sensations had changed in every subject despite no significant changes in cortical reorganization. The results of this study suggest that pain sensation and perception are highly malleable and may not be causally related to cortical reorganization.

Adult-onset CRPS is clinically distinct from childhood-onset CRPS. Unlike in adult-onset CRPS, children with CRPS usually experience full resolution of pain symptoms (Low et al., 2007; Linnman et al., 2013; Weissmann and Uziel, 2016). One study examining childhood-onset CRPS found altered functional connectivity in five key brain structures: amygdala, ACC, caudate, post-central gyrus, and putamen (Linnman et al., 2013). Interestingly, the researchers observed that CRPS children with complete pain resolution still exhibited altered functional connectivity in these brain structures. Similar to cortical reorganization, then, altered functional connectivity and pain perception are not causally connected. Unfortunately, their analysis did not examine functional connectivity of mPFC, a region that would be of interest for pain perception and its relation to conditioned fear. Future studies should examine structural and functional connectivity between mPFC and limbic regions to address the question of whether pain perception in CRPS is related to neural circuitry of fear conditioning, and is perhaps a conditioned phenomenon.

Mirror therapy has been shown to be an effective therapeutic modality for phantom limb pain (Chan et al., 2007; Finn et al., 2017), while motor imagery therapy has been documented to be an effective treatment for CRPS (Moseley, 2004b; Bowering et al., 2013). Treatments targeting motor movement may help to regulate altered cortical representations of the affected limb in the primary motor cortex and S1. In addition, such therapeutic modalities may be effective through changes in proprioceptive representations of limb positioning and body movement within the dorsal visual processing stream, or “where” visual pathway (Preissler et al., 2013). In addition to changes within primary motor cortex, S1, and posterior parietal areas, mirror and motor imagery therapy may also facilitate the growth of new neural circuits that inhibit previously established fear-associated connections. From a classical conditioning standpoint, these therapies may lead to the formation of new CS-US pairings, in which the proprioceptive feedback resulting from the therapy (the CS) is associated with the pain-free state (the US). The CR, then, would be the relief state resulting from being pain free. Through classical conditioning, additional proprioceptive representations can serve as neutral stimuli to become associated with a pain-free state, thereby inhibiting the original pain associations.

## Chronic Back Pain

As with phantom limb pain and CRPS, chronic back pain (CBP) is associated with central sensitization (Woolf, 2011; Roussel et al., 2013). In addition, CBP results in structural and functional changes in attentional, emotional, and default mode networks (Apkarian et al., 2004; Baliki et al., 2006, 2008; Tagliazucchi et al., 2010; Seminowicz et al., 2011; Hashmi et al., 2013; Zhang et al., 2014). Specifically, CBP is associated with a 5%–11% reduction in overall cortical volume, with symptom duration correlated with amount of gray matter loss (Apkarian et al., 2004). In addition, reduced gray matter density in dlPFC correlates with increased pain severity, while increased dlPFC thickness following surgery or other intervention procedures correlates with a reduction in reported pain (Apkarian et al., 2004; Seminowicz et al., 2011). These studies, however, did not differentiate the temporal divide between pain resolution and increase in dlPFC thickness. Thus, one cannot conclude whether symptoms decreased as a result of increased cortical gray matter density or whether patients achieved pain-free states prior to cortical reorganization. Similar results have been found in hip osteoarthritis (coxarthrosis; Rodriguez-Raecke et al., 2013), such that pain resolution preceded increased dlPFC thickness. Therefore, the relationship between dlPFC thickness and chronic pain may be merely correlational, or perhaps, pain resolution leads to alterations in cortical circuitry.

Interestingly, Hashmi and associates (Hashmi et al., 2013) noted a functional difference between CBP and acute back pain, classified as back pain occurring for less than 3 months. While experiencing increased levels of pain, subjects with acute back pain consistently showed increased activations in the ACC and insula, two key structures within the salience network. Individuals with CBP, however, showed increased activations in the mPFC and amygdala. The results of this study highlight the role of ACC and insula in initial pain detection. In another study, patients with CBP exhibited increased pain levels due to thermal stimulation, and their reported pain levels were positively associated with activation in insular cortex (Baliki et al., 2006). Spontaneous pain in these patients, however, was correlated with activation in mPFC. Thus, in CBP, pain perception in the absence of an overt threatening or noxious stimulus seems to be facilitated through mPFC. It is possible that when acute pain becomes chronic, the mPFC and fear associated structures may begin to play an important mediating role in the perception of pain. Further, dysregulation within this region may help to explain the altered dynamics within DMN observed in patients with CBP (Baliki et al., 2006, 2008; Hashmi et al., 2013).

Why do some individuals present with back pain while others with similar physiological findings do not? Perhaps subjects who do not report back pain are less likely to form pain-related fear whenever a back condition occurs. Alternatively, perhaps pain-free individuals with back bulges do not experience pain because the brain does not detect the disc bulge as being a threat. In a standard fear-avoidance model, pain-related fear involves threat perception (Leeuw et al., 2007). Perhaps the asymptomatic subjects with disc bulges have little to no pain-related fear, which explains their lack of symptoms. This model, however, is speculative and future research is necessary to provide further support for the idea that pain-related fear avoidance is causally associated with verbal report of pain symptoms.

Motor control therapy has been found to be a successful form of treatment for CBP (Macedo et al., 2012; Saragiotto et al., 2016). Similar to therapies for phantom limb pain and CRPS, motor control therapy may help to form new CS-US relationships that may inhibit or alter pre-existing pain and fear associations. Support for this theory comes from prior work showing that in conjunction with motor control therapy, pain physiology education courses can help decrease pain scores in CBP (Moseley, 2004a; Moseley and Butler, 2015). How can these education courses help to inhibit pain and fear associations? From a cognitive-learning perspective, maladaptive thoughts such as, “something must be seriously wrong with me, this pain is indicative of something worse,” may become the CR in response to pain. As discussed earlier, cognitive representations of pain may also serve as an antecedent CS leading to the CR of increased anxiety. Through cognitive remediation, an individual can reframe these thoughts in a more adaptive manner: “I am in pain right now, but from a physiological perspective, I am not in any life-threatening danger.” This cognitive reappraisal may then reduce pain-related fear and pain-related anxiety by preventing negative reinforcement, which in turn will reduce avoidance behaviors.

## An Integrative Mind-Body Approach to the Treatment of Chronic Pain

The molecular mechanisms underlying central sensitization involve glutamate and non-glutamate receptors (Figure 1), and are similar in nature to those of LTP in fear-related circuits. Currently, treatments seeking to alter the molecular mechanisms of central sensitization, such as NMDAR antagonists and nociceptive input, are not universally successful in treating different chronic pain syndromes. Similarly, previously observed cortical and functional alterations have not been causally related to chronic pain. Further, pain resolution in chronic pain syndromes may occur even if cortical and functional alterations are still apparent. Rather than focus on altered cortical thickness or connectivity, we suggest a greater emphasis be placed upon how structures communicate with one another, particularly how the mPFC communicates with other brain structures in the salience network, fear network and the DMN. As demonstrated in patients with CBP, the dlPFC plays a regulatory role in pain perception and may provide inhibitory pathways that suppress pain and fear circuits. However, given the mPFC’s connection to the DMN and fear circuit, along with its projections to the salience network, this brain structure may be of greater importance for pain modulation. Recent pre-clinical evidence indicating that vmPFC activity is required for the effectiveness of extinction-based therapies (Fucich et al., 2018) buttresses the importance of this structure in therapeutic success.

**Figure 2:**
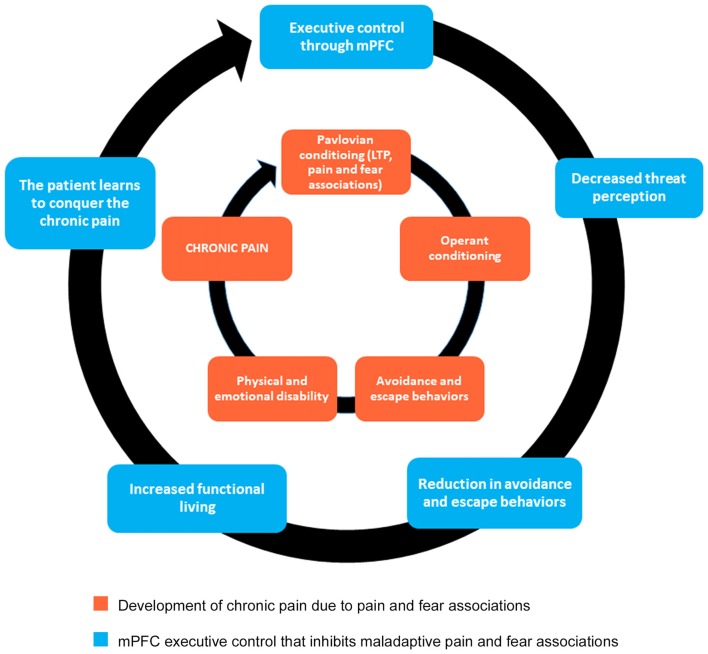
Mind-body approach to healing that promotes executive control originating from mPFC. The inner circle indicates the behavioral mechanisms underlying chronic pain. This approach to healing, grounded in cognitive reappraisal, mindfulness meditation, and functional rehabilitation, will promote new synaptic connections necessary for fear extinction (outer circle). Notice that the inner circle does not go away. Instead, by strengthening the components of the outer circle, the mPFC can exercise executive control by inhibiting maladaptive pathways (inner circle). As a result, chronic pain patients learn how to better cope with pain, in essence giving them the power to conquer the debilitating nature of their pain.

Therefore, to more successfully treat chronic pain conditions, we propose an alternative approach to psychotropic agents that downregulate glutamate and non-glutamate receptors. Instead of attempting to reverse central sensitization occurring at the level of the spinal cord, we suggest the use of a cognitive behavioral program that will foster the development of new synaptic connections between the mPFC and other structures that will, in turn, inhibit LTP within pain and fear circuits (Figure 2). We further propose that the most effective form of chronic pain rehabilitation is through a combination of cognitive reappraisals, mindfulness meditation, and functional rehabilitation (Figure 2). As previously mentioned in the discussion of CBP, cognitive reappraisals may help to reduce pain-related fear and anxiety, which may then reduce avoidance behaviors (see Table 1 for a list of cognitive reappraisals). Indirect evidence exists indicating that cognitive reappraisal can modify central sensitization processes, as cognitive behavioral therapy (CBT) emphasizing pain regulation strategies has been shown to reduce secondary hyperalgesia (Salomons et al., 2014). Evidence also indicates that therapy grounded in cognitive reappraisal and the reduction of catastrophic thoughts increases functional brain connectivity between S1 and anterior insula (Lazaridou et al., 2017) and between the DMN and executive control networks (Kucyi et al., 2016). CBT has also been shown to normalize aberrant functional connectivity in frontal-parietal attentional circuitry in chronic pain patients, with higher connectivity associated with greater reduction in pain intensity ratings (Yoshino et al., 2018). Similarly, in chronic pain patients, CBT has been shown to increase gray matter volume in lateral PFC, the pregenual region of ACC, posterior parietal cortex and somatosensory cortex, accompanied by a reduction in catastrophizing (Seminowicz et al., 2013). Thus, cognitive therapies for pain management have the potential to alter neural circuits related to attention, emotion, and the integration of pain signals. Hence, we hypothesize that cognitive reappraisals may promote the reduction of pain and the extinction of conditioned pain-related fear through top-down regulation of brain regions involved in emotion and sensory processing. This process may be mediated by a general reduction in the negative affective states associated with pain perception.

**Table 1:** Examples of turning maladaptive cognitions into adaptive cognitive reappraisals.

Maladaptive cognitions	Cognitive reappraisals
This pain will never go away.	I am in pain right now but that does not mean this pain will last forever.
There is no definitive cure. I will never be pain-free.	Advances are continually made in medicine and a cure may be found in the future.
I do not want to engage in any activity because I am in pain.	Even though I am in pain, let me see what I can do within a reasonable limit without causing any further pain.
I am a victim. If doctors cannot fix me, I cannot fix myself.	I am an active participant in my recovery.

Unlike cognitive reappraisals, mindfulness meditation focuses on present moment awareness without becoming emotionally involved or overtaken with sensations or thoughts (Kabat-Zinn et al., 1985). Studies have demonstrated small to moderate treatment effects for both mindfulness meditation and mindfulness-based stress reduction (MBSR) for chronic pain and related conditions (Crowe et al., 2016; Hilton et al., 2017), and it appears as if change in mindfulness scores mediates better clinical outcomes (Alsubaie et al., 2017). Neuroimaging evidence indicates that reductions in pain severity accompanying MBSR for chronic pain are associated with increased functional connectivity between anterior insula and ACC (Su et al., 2016). Similarly, among mindfulness meditators, reductions in pain unpleasantness ratings accompanying a noxious stimulus have been associated with decreased activation in lateral PFC and increased activation in posterior insula (Gard et al., 2012). These authors also observed an increase in ACC activation during the anticipation of a painful stimulus accompanied by a reduction in anticipatory anxiety among the mindfulness meditators, but not controls. Further, in a direct comparison of the neural mechanisms of mindfulness to those of “sham” mindfulness, Zeidan and associates (Zeidan et al., 2015) observed that reduced pain intensity and unpleasantness ratings to a painful stimulus were associated with increased activation in subgenual ACC, orbitofrontal cortex and anterior insula during mindfulness, but not sham mindfulness. Additional studies have shown that mindfulness meditation is associated with reduction in anxiety in both clinical (Marchand, 2012) and nonclinical (Zeidan et al., 2014) populations, and compared with non-meditating subjects, mindfulness meditation increases co-activation of the mPFC and ACC (Brown and Jones, 2010). As previously mentioned, this co-activation is associated with fear appraisal (Maier et al., 2012).

Increased co-activation of mPFC and ACC in mindfulness meditators may be indicative of the mPFC’s top-down influence, or executive control, on the salience network. Further, if the salience network is involved in threat perception, the mPFC can deem the sensation of pain as non-threatening, which helps to explain pain reduction in mindfulness meditation (Grant and Rainville, 2009; Zeidan et al., 2011). By acting on the mPFC, mindfulness meditation can also help to normalize dysregulation within the DMN (Brewer et al., 2011). Importantly, evidence suggests that mindfulness meditation results in pain reduction through its effects on pain circuits rather than through placebo effects (Zeidan et al., 2015). Recent evidence also suggests that sleep deprivation may increase pain sensitivity and reduce the effectiveness of pain-reducing medication (Alexandre et al., 2017). Therefore, methods that help promote relaxation and enhance sleep quality may be useful additions to a mindfulness meditation or MBSR program for the treatment of chronic pain.

Lastly, functional rehabilitation aims to increase patient range of motion and functional movements in daily living, such as walking, squatting and bending over. Similar to mirror therapy in phantom limb pain, motor imagery therapy in CRPS, and motor control in CBP, functional rehabilitation will also form new CS-US relationships which may then be generalized to other stimuli. We emphasize here that functional rehabilitation does not promote mind over body. Therefore, if functional movement exacerbates the pain symptoms, a chronic pain patient should not try to push through the pain in that moment. Instead, chronic pain patients should carefully assess for pain by trying different functional movements and slowly increase movement or range of motion when pain is not prohibitive (Ambrose and Golightly, 2015).

Because complete pain reduction is not always feasible, it is critical that the chronic pain patient does not use pain resolution as a barometer for success, as continued pain and fear associations may become barriers to success. Instead, increased functionality in daily living should become the barometer. Hence, the goal of our suggested approach to treating chronic pain is not to eliminate the pain entirely, but to conquer the maladaptive cognitive appraisals and the established neural associations in fear circuitry that make it difficult to function. Such a comprehensive treatment plan should lead to increased success rates for chronic pain interventions. When necessary and where indicated, medication management should accompany these therapeutic programs. However, a combination of the three non-pharmacological therapies should mitigate the need for pharmacological intervention, and perhaps, eliminate it altogether over time.

## Conclusion and Protocol Recommendation

In conclusion, we offer the hypothesis that the most effective and beneficial treatment program for long-term management of chronic pain requires three unique aspects of therapy: (1) cognitive reappraisals; (2) mindfulness meditation; and (3) functional rehabilitation. We further hypothesize that a multimodal treatment program will lead to increased connectivity between mPFC and other cortical/subcortical regions, such as insula and amygdala, compared with any one of these therapies alone. To test this hypothesis, we suggest the implementation of an 8- to 12-week four-arm randomized clinical trial for patients experiencing chronic pain. To attain sufficient power for detecting differences among groups, 120 patients should be randomized to one of the following conditions: CBT alone, mindfulness meditation alone, functional rehabilitation alone, or combined multimodal therapy including CBT, mindfulness and functional rehabilitation. The randomization process should lead to approximately 30 patients per group, which is an adequate sample size even with the likelihood of patient drop-out and issues with image acquisition, such as participant motion or other artifact.

For CBT, we recommend a program consisting of weekly 45-min sessions comprised of: psychoeducation regarding the learned mechanisms of avoidance, cognitive restructuring through reappraisal, attention diversion and self-regulatory skills. Although prior studies have utilized a group approach to CBT (Seminowicz et al., 2013; Yoshino et al., 2018), we recommend individually-tailored CBT, if feasible, to maximize effectiveness. For mindfulness, we recommend a modified protocol based upon prior work (Zeidan et al., 2011, 2015). Weekly 45-min sessions with the therapist should include a 30-min mindfulness program comprised of: following the breath, progressive body scan and nonjudgmental awareness of thoughts. The therapist conducting these sessions should intentionally instruct the participant to become aware of body sensations, teaching the participants to allow these sensations to arise without judgment or emotional reaction. For functional rehabilitation, we suggest twice weekly 45-min sessions encompassing a program of walking, strength training, and stretching exercises (Lee and Kang, 2016), including walking outdoors or on a treadmill, depending on feasibility. Because it would not be feasible to incorporate full versions of these different therapies into a program for the combined therapy group, the multimodal treatment program should include weekly 90-min sessions incorporating abbreviated aspects of each of the three individual therapies. For each session, we recommend 30 min of CBT, followed by a 20 min mindfulness session, and then 40 min of functional rehabilitation.

To examine putative changes in functional brain connectivity, along with regional brain activity associated with monitoring painful sensations, study participants should be scanned twice: once prior to the initiation of assigned therapy and once following the conclusion of the intervention. Scanning protocol should include a 5-min “eyes-closed” resting state fMRI series, followed by a simple block-design fMRI procedure that includes alternating blocks of maintaining fixation on a central fixation cross and actively monitoring potentially painful body sensations. Changes in structural connectivity can be assessed by including a diffusion tensor imaging (DTI) scanning series, if available.

We acknowledge the ambitious nature of this proposed clinical trial, requiring many hours of therapeutic services and a total of 240 MRI scanning sessions. Therefore, we recommend a multi-site approach to this intensive investigation. Despite the likelihood that such a study might take a few years to complete, the knowledge gained through this study will have lasting impacts on treatment recommendations for chronic pain.

## Author Contributions

JG conceived the primary hypothesis put forward in this article. Both JG and KS contributed to searching prior literature, and both authors contributed to the writing and editing of this manuscript.

## Conflict of Interest Statement

The authors declare that the research was conducted in the absence of any commercial or financial relationships that could be construed as a potential conflict of interest.
